# Psychiatric readmission of children and adolescents in China: a scoping review

**DOI:** 10.3389/fpsyt.2026.1760740

**Published:** 2026-03-04

**Authors:** Wanqi Sun, Qiqi He, Chenghaoran Wu, Hao Yao, Wenqing Jiang

**Affiliations:** Shanghai Mental Health Center, Shanghai Jiao Tong University School of Medicine, Shanghai, China

**Keywords:** children and adolescents, China, mental health, psychiatric readmission, scoping review

## Abstract

**Objectives:**

Psychiatric readmission in children and adolescents is not uncommon in developed countries. Despite increasing medical resources being allocated to children and adolescents with mental health problems in China, the effectiveness of psychiatric inpatient treatment and the current situation of rehospitalization are not well documented.

**Methods:**

A scoping review was conducted to describe the actual prevalence, associative factors and potential interventions for psychiatric readmission among children and adolescents in China. Documents indexed in PubMed, PsycINFO, Web of Science, Wanfang and CNKI were searched for up to May 2025. Two authors independently reviewed the records.

**Results:**

Of the 723 records, 19 met the inclusion criteria. For children and adolescents with heterogeneous mental health diagnoses, the weighted average readmission rate was 18.6% overall, with rates of 8.6% for follow-up periods under one year and 20.9% for periods of one year or more, respectively. The readmission rate was higher in patients with schizophrenia spectrum disorders and lower in those with mood disorders. The risk factors related to psychiatric readmission included longer follow-up duration and hospitalization of the initial treatment, poor adherence to medication, frequent changes in treatment regimens, poor treatment responses, childhood onset, family history of mental illness, excitability/hostility, insufficient sleep, anxiety, and obesity. The protective factors related to psychiatric readmission included support from family, teachers and friends for patients with depression, and obsessive–compulsive symptoms for patients with schizophrenia. Only preliminary evidence was found regarding interventions to reduce readmission for pediatric psychiatric inpatients or their parents.

**Conclusions:**

This review provides a significant overview of current psychiatric inpatient treatments for children and adolescents in China. Our findings underscore the notable frequency of psychiatric readmissions, highlighting the need for targeted interventions, particularly for children and adolescents exhibiting certain clinical features and challenges with treatment adherence or responsiveness.

## 1 Introduction

Readmissions in children and adolescents following inpatient psychiatric treatment are not uncommon. A previous meta-analysis reported that 13.2% of youth were readmitted to psychiatric hospitals, and the mean time to readmission was approximately 13 months (1). Repeated admissions might lead to more severe social impairments in children and adolescents and increased health economic costs. Adolescents who experienced psychiatric inpatient episodes were twice as likely as their peers to be not in education, employment, or training (NEET) when they entered the labor market (2). Moreover, according to data from the Global Burden of Disease (GBD) Study, youth aged 5–24 years account for approximately one-fourth of the mental disorder burden across the entire life course (3). Mental disorders are also the leading cause of disability-adjusted life years (DALYs) among children and adolescents in China (4). Furthermore, mental disorders that begin in childhood or adolescence are usually more difficult to treat and carry a higher risk of readmission. Compared with both nonreadmitted patients and matched controls, readmitted patients, particularly those with schizophrenia, experience a significantly higher mean frequency of care and greater medical costs (5). Therefore, it is important to reduce readmissions to achieve a cost-effective balance, as inpatient mental health treatment resources are limited and costly in China.

The emergence of mental health problems in children and adolescents is associated with multifaceted risk factors (6), and these factors may also influence the course and prognosis of these problems. The potential factors related to psychiatric readmission may include the patient’s individual conditions (e.g., diagnosis, symptom severity and comorbidity), family support systems, the capabilities of healthcare institutions, the quality of treatment processes (e.g., routine of admission and after-care), as well as contextual factors (e.g., regional economy, policies and community resources) (7–10). A significant number of factors within this scope differ greatly between China and Western countries, especially those describing family, hospital and broader contextual features. Thus, it is imperative to investigate the factors influencing the readmission of children and adolescents to psychiatric care within the distinctive social and cultural context of China. This understanding will facilitate the development of more targeted interventions.

Readmission rates, particularly short-term readmissions (e.g., within 30 days of discharge), serve as a key indicator of healthcare quality (11). This encompasses the quality of inpatient care, discharge planning and transition, as well as postdischarge aftercare. The benefits of successful initial treatment could be twofold. On the one hand, initial interventions that effectively reduce the duration of first episodes of mental health problems in children and adolescents may significantly decrease the probability of recurrence during young adulthood (12). On the other hand, higher levels of satisfaction with inpatient care are associated with an increased likelihood of continuing outpatient visits postdischarge, thereby potentially reducing the rate of readmission (13). Experience from Europe suggests that continuity of care (i.e., having the same clinicians responsible for a patient’s care across inpatient and outpatient settings) could reduce the number of admissions (14). Unfortunately, such care is not always supported by the current Chinese medical system, making discharge planning and transition programs important. Discharge interventions for child and adolecent inpatients, including risk assessment, individualized care, discharge preparation, community linkage, psychoeducation, and follow-up support, effectively minimize patient and family vulnerability postdischarge and thereby reduce the risk of readmission (15). Moreover, receiving aftercare services within one month postdischarge is associated with lower readmission rates in a dose–response manner (16, 17). However, it remains uncertain whether a service coordination mechanism for the inpatient and outpatient care of children and adolescents admitted to psychiatric wards, as well as an evidence-based postdischarge program, exists in China.

Since the population of children and adolescents is substantial and mental health resources are evolving in China, understanding the patterns and determinants of psychiatric readmission is crucial for improving care and outcomes. However, current knowledge of psychiatric readmission for children and adolescents in China is not well documented. The purpose of this study was to (a) describe the prevalence of psychiatric readmission, (b) identify the risk and protective factors for psychiatric readmission, and (c) identify intervention programs aimed at reducing psychiatric readmission among children and adolescents with mental health problems in China.

## 2 Methods

This study employed the scoping review methods recommended by Arksey and O’Malley (18) and Levac, Colquhoun, and O’Brien (19). The results were reported via guidelines provided by the PRISMA extension for scoping reviews (20).

### 2.1 Eligibility criteria

Studies were eligible for inclusion if they were 1) published in English or Chinese, 2) were particularly related to readmission to a psychiatric ward or clinical psychology unit, and 3) included patients under 19 years of age according to the World Health Organization (WHO)’s definition of children and adolescents. The year of publication was not restricted. The studies were excluded if they did not provide separate discussions or results for children and adolescents, even if the overall sample included some participants aged 18–19 years.

### 2.2 Search strategy

Searches were conducted in three English databases (i.e., Web of Science, PubMed, and PsychINFO) and two Chinese databases (i.e., Wangfang and CNKI). The search terms included (psych* OR mental health) AND (readmi* OR rehosp*) AND (adolesc* OR child* OR youth) AND (China OR Chinese). The search terms were translated and refined into Chinese. The detailed search terms used in each database are shown in the **Supplementary Material**.

### 2.3 Study selection

Two researchers each conducted separate screenings of the titles and abstracts from the search results to generate a list of studies for comprehensive review. Any study identified as potentially eligible by at least one reviewer was retrieved for full-text assessment. Both researchers independently examined the full texts of these identified studies to decide on their inclusion. Any discrepancies in inclusion decisions during this stage were resolved through team deliberation.

### 2.4 Charting the data

Included studies were then subjected to a structured data extraction procedure in which the following information was recorded and charted: author and publication year, language, article type, study location, study design, research period, sample characteristics, main questions of interest mentioned by the article, and summary of findings related to readmission.

### 2.5 Collating, summarizing, and reporting results

The findings were synthesized and presented mainly through a narrative approach. This method included categorizing studies based on three main questions of interest: (a) the prevalence of readmission, (b) factors associated with readmission, and (c) interventions aimed at reducing readmission. The key results from the studies were analyzed, compared, and synthesized to highlight recurring themes observed across multiple investigations. A weighted average (i.e., Σ(rate × sample size)/total N) was calculated for the readmission rate where sufficient data were available, and the results were stratified by follow-up duration and diagnosis. We used the Mixed Methods Appraisal Tool to assess the methodological quality of the included studies (21). The critical appraisals were independently evaluated by two researchers. Discrepancies were resolved by discussion until a consensus was reached.

## 3 Results

**Figure 1:**
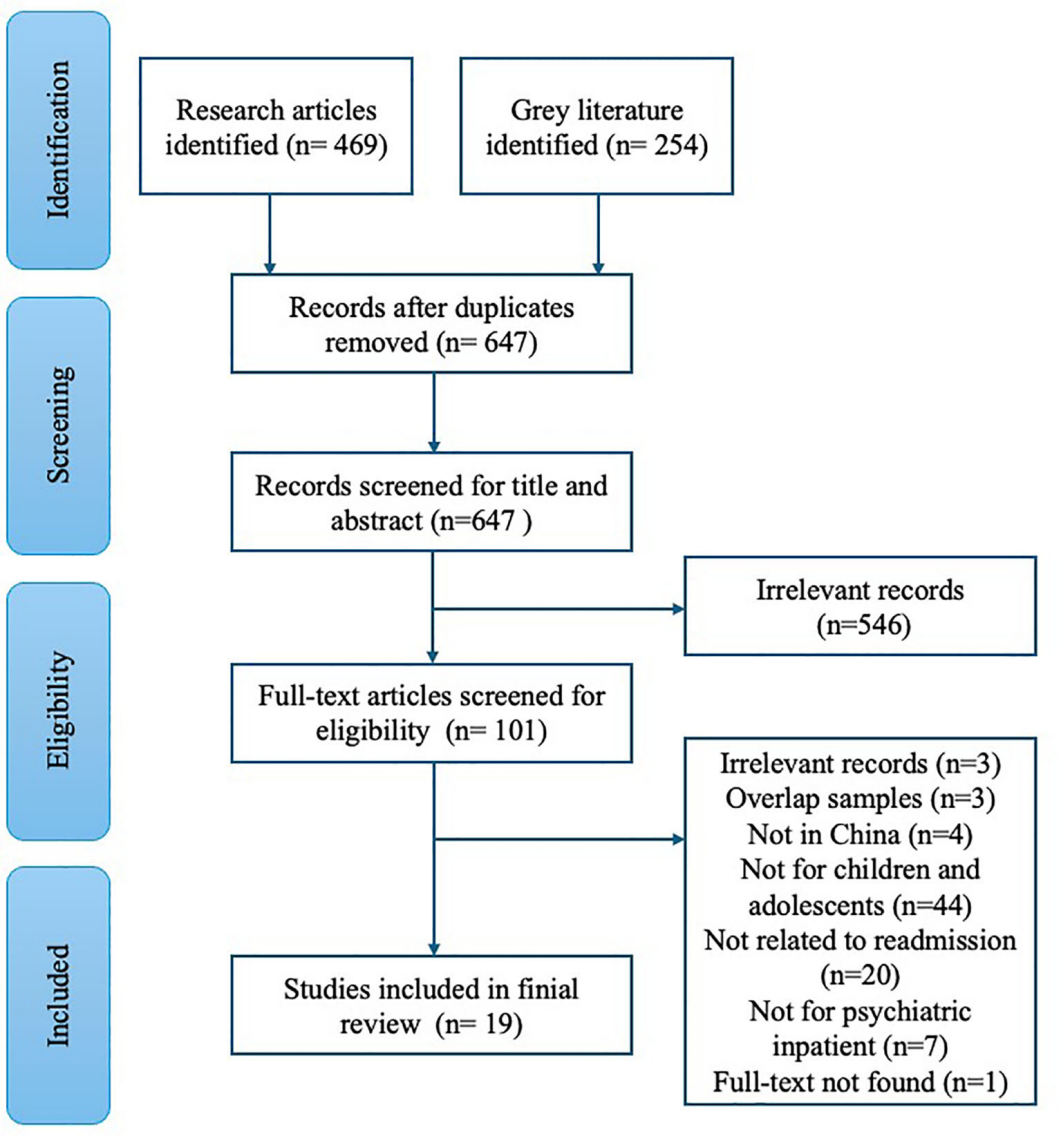
Flow diagram of the scoping review selection process.

Of the 723 records, 19 met the inclusion criteria (**Figure 1**). An initial search identified 469 original articles and 254 gray literature (e.g., theses, conference abstracts, patents, and research achievements). Six hundred and forty-seven nonduplicate records were screened based on title and abstract. Five hundred and forty-six irrelevant records were excluded during the title and abstract review. One hundred and one records underwent full-text review. Forty-four records were excluded because the samples did not include children and adolescents, 20 were unrelated to readmission, 7 were not about psychiatric inpatients, 4 were not conducted in China, 3 studies used overlapping samples, 3 records were beyond the scope of this review, and 1 full-text could not be found. Seventeen of the included recordings were research articles, one was a conference abstract, and one was a master’s thesis.

### 3.1 Critical appraisal

Three studies were randomized controlled trials, but the outcome assessors were blinded only in one study. Two were nonrandomized intervention studies, but confounders were not accounted for. Fourteen studies reported a quantitative descriptive design that adopted appropriate measurements and statistical analysis methods. Eleven of these studies clarified the inclusion and exclusion criteria for the participants, and ten studies were regarded as having a low risk of nonresponse bias. All studies reported data collected from a single medical center. Details of the critical appraisal are shown in the **Supplementary Table**.

### 3.2 The prevalence of psychiatric readmission among children and adolescents

Thirteen studies reported the prevalence of psychiatric readmission among children and adolescents. Of the included studies, nine were retrospective and four were prospective longitudinal, with follow-up durations ranging from 30 days to 6 years. The extracted readmission rates across diagnostic groups are listed in **Table 1** and a narrative synthesis of the review results is provided in **Table 2**.

**Table 1:** The prevalence of readmission across diagnostic groups.

Author, year	Prevalence (%)	Sample size (N)	Follow-up period
Heterogeneous			
Yue, 2017 (22)	65.9	82	/
Li, 2018 (23)	13.6	550	/
Huang, 2020 (24)	19.7	2096	/
Li, 2021 (25)	19.5	3940	1-yr
Schizophrenia			
Chen, 1976 (26)	41.0	100	/
Chen, 2002 (27)	42.9	76	/
Yue, 2017 (22)	66.7	36	/
Yang, 2017 (28)	58.5	81	5-yrs
Zhang, 2018 (29)	56.7	83	5-yrs
Huang, 2020 (24)	21.7	1430	/
Zheng, 2021 (30)	25.9	276	6-yrs
Mood disorders			
Yue, 2017 (22)	81.8	22	/
Huang, 2020 (24)	18.5	357	/
Zhu, 2022 (31)	4.9	1107	30-d
Deng, 2023 (32)	17.6	348	1-yr
Developmental disorders			
Yang, 2024 (33)	7.0/18.8	341	31-d/1-yr
Huang, 2020 (24)	18.5	357	/

**Table 2:** Characteristics of included studies reporting readmission prevalence and associated factors.

Reference (Author, year)	Language	Article type	Study design	Research period	Sample characteristics	Main findings related to readmission
Chen, 1976 (26)	Chinese	Original article	Retrospective analysis from medical record	1963-1974	100 schizophrenia patients with first onset age of 12-17-yrs	Among the 100 adolescent schizophrenia patients, 59 were hospitalized once, 32 were hospitalized twice, and 9 were hospitalized three times. The first hospitalization required a relatively short course of treatment. However, during the second and third hospitalizations, the length of hospital stay increased, medication dosages were escalated, and treatment effectiveness progressively declined.
Chen, 2002 (27)	Chinese	Original article	Retrospective analysis from medical record	1993-2000	76 children under 16-yrs old with schizophrenia	Of the 76 patients, 40 were hospitalised once, 23 twice and 7 three times.
Zhang, 2018 (29)	Chinese	Original article	Prospective longitudinal study	2012-2018 (5-yr follow-up: 3- mo, then annually)	83 schizohprenia inpatients under 14-yrs old	During the 5-yr follow-up, 38 (56.7%) children had readmissions. From the first to the sixth follow-up, the number of readmissions was 9 (13.4%), 16 (23.9%), 19 (28.4%), 15 (22.4%), 12 (17.9%), and 10 (14.9%). Among them, 4 children had 4 readmissions, 5 children had 3, 21 children had 2 and 8 children had 1. The time to readmission ranged from 34 to 352 d, with a median interval of 196 d.
Li, 2018 (23)	Chinese	Original article	Retrospective analysis from medical record	2014-2017	550 psychiatric inpatients under 18-yrs old	Compared with the adolescent onset group (age of onset: 13-18-yrs old), patients in the childhood onset group (age of onset ≤ 13 years) had a higher rate of readmission (18.3% vs. 11%, P = 0.018).
Huang, 2020 (24)	Chinese	Master thesis	Retrospective analysis from medical record	2012-2018	2,096 psychiatric inpatients under 18-yrs old	The overall readmission rate was 19.7%. Specifically, the readmission rate for schizophrenia spectrum disorders was 24.8%, followed by mood disorders at 18.5%. The re-admission rate (≥2 admissions) for the childhood-onset group (onset age ≤13 years) was higher than that for the adolescent-onset group.
Li, 2021 (25)	Chinese	Original article	Retrospective analysis from medical record	2013-2017	22,807 psychiatric inpatients (3,940 patients under 20-yrs old)	In the whole sample, 20.2% of patients were readmitted within 1 year of hospital discharge, while the readmission rate for youth under 20-yrs-old was 19.5%. Patients under 20 had a higher rate of readmission within one year of discharge than those aged 21 to 60.
Zhu, 2022 (31)	English	Original article	Retrospective analysis from medical record	2009-2018	13,177 patients with major depressive disorder (1,107 patients under 17-yrs old)	The 30-day readmission rate for youths under 17-yrs old was 4.9%, higher than that for adults aged 18-60-yrs old (3.0%). This study developed a prediction model for readmission based on sociodemographic and clinical features, symptoms at admission, and treatment during the index admission.
Lai, 2024 (8)	English	Original article	Retrospective analysis from medical record	2021-2022	49,352 patients with mental and behavioural disorders, 7,814 children and adolescents aged 0-17-yrs	In the overall sample, the 31-day unplanned readmission rate was 8.6%. Compared with patients aged 65 and above, aged 17-yrs patients and younger had a lower readmission rate. Associated factors for children and adolescents were not examined specifically.
Yang, 2017 (28)	Chinese	Original article	Prospective longitudinal study	2008-2015 (5-yr follow-up)	81 youths with schizophrenia under 18-yrs old (The number of valid cases is 68)	The readmission rate was 58.5%. The highest number of hospitalizations was 5 times in 4 cases, 4 times in 6 cases, 3 times in 11 cases, and 2 times in 19 cases. A high Excitability Hostility Factor score at baseline, poor adherence to medication and a high number of changes in treatment regimen were risk factors for readmission.
Yue, 2017 (22)	Chinese	Conference abstract	Retrospective analysis from medical record	Half year in 2016-2017	82 psychiatric inpatient children and adolescents	There were 28 patients (34.1%) were admitted for the first time, while 54 (65.9%) were readmitted. Among the readmitted patients, 77.8% of the patients were readmitted due to self-initiated discontinuation or reduction of medication on their own.
Zheng, 2021 (30)	English	Original article	Prospective longitudinal study	2000-2018 (6-yr folow-up)	276 patients with schizophrenia, age of first onset ≤14-yrs old	Forty-four patients (25.9%) were readmitted during the follow-up period. Longer follow-up duration was risk factors for readmission, while the presentation of obsessive-compulsive symptoms was protective factor.
Deng, 2023 (32)	Chinese	Original article	Prospective longitudinal study	2018-2022 (1-yr follow-up)	348 children and adolescents aged 14-18-yrs old with first onset depression	The 1 year readmission rate was 17.62%. BMI ≥ 24 kg/m2, family history of mental disorder, lack of sleep and anxiety were risk factors for readmission, while the total score and the scores for family support, teacher support and friend support on the Multidimensional Scale of Perceived Social Support Scale were protective factors for readmission.
Yang, 2024 (33)	Chinese	Original article	Retrospective analysis from medical record	2022	341 patients with autism aged 3-17-yrs	The 31-day and 1-year unplanned readmission rates were 7.0% and 18.8%. Children aged 3-6-yrs had a higher 31-day unplanned readmission rate compared to older children aged 7-17-yrs. Patients with a longer initial hospitalization of over 15 days had higher 31-day and 1-year unplanned readmission rates than those with a shorter initial hospitalization.
Si, 2022 (34)	English	Original article	Retrospective analysis from medical record	2015-2020	410 patients aged 14–18 yrs with schizophrenia, major depressive disorder, or bipolar disorder who received MECT treatment	At the end of the follow-up, the readmissions rate was 20.49%. A significantly higher proportion of readmission was found in BD non-responders than in responders (19.5% vs. 34.5%, P = 0.029), there was no difference in the rate of readmission in the other diagnostic groups.

The readmission rate for children and adolescents across heterogeneous diagnostic groups (N = 12, total subjects: 9080) ranged from 4.9% (31) to 65.9% (22), with a weighted average rate of 18.6%. Three studies reported the readmission rate for follow-up or research periods of less than one year across various diagnoses, with a weighted average of 8.6%. Six studies reported the rate for periods of at least one year, with a weighted average of 20.9%. Another retrospective quantitative analysis conducted among patients with mental and behavioral disorders reported that compared with those aged 65 and above, patients younger than 17 years old had a lower 31-day unplanned readmission rate (8). However, the specific readmission rate for each age group was not reported.

The readmission rate for children and adolescents diagnosed with schizophrenia (N = 7, total subjects: 2082) was high, ranging from 21.7% (24) to 66.7% (28), with a weighted average rate of 27.6%. Two studies adopted a prospective design and suggested that the readmission rate was associated with the follow-up duration. One study showed that a longer follow-up duration was related to higher accumulated readmission (30). The other 5-year longitudinal study reported the time to readmission ranged 34–352 days, with a median interval of 196 days. At three months, 1 year, 2 years, 3 years, 4 years, and 5 years after discharge, the percentages of readmissions were 13.4%, 23.9%, 28.4%, 22.4%, 17.9%, and 14.9%, respectively (29).

Two studies reported the readmission rate for children and adolescents diagnosed with depression (N = 2, total subjects: 1445). In a large-scale retrospective study of patients with major depressive disorders, the 30-day readmission rate of youths under 17 years of age was 4.9%, higher than that of adults aged 18–60 years (3.0%) (31). In a prospective study, the 1-year readmission rate was 17.6% (32). According to a separate retrospective study focusing on mood disorders (ICD-10 F30-F39), the readmission rate was 18.5% (24). One conference paper reported the readmission rate in a very small sample (N = 22) of children and adolescents with bipolar disorder as high as 81.8% (22). A weighted average rate was not computed because the studies had different observation periods and heterogeneous samples.

Two studies reported on the readmission rate of children and adolescents with developmental disorders. Yang and Zhao reported data on children and adolescents with autism and found that the 31-day and 1-year unplanned readmission rates were 7.0% and 18.8%, respectively (33). Huang reported a readmission rate of 14.1% for children and adolescents diagnosed with intellectual disability (ICD-10 F70-F79), without defining the observation duration (24).

Readmission rate for other diagnose were less studied. Huang (24) also reported the readmission data for other ICD-10 diagnosis categories 16.0% for F00-F09 (organic mental disorders), 11.1% for F40-F48 (neurotic, stress-related, and somatoform disorders), 9.0% for F90-F98 (behavioral and emotional disorders with onset usually occurring in childhood and adolescence) (24).

### 3.3 The risk and protective factors for psychiatric readmission

The risk factors related to psychiatric readmission included longer initial hospitalization period, poor adherence to medication, frequent changes in treatment regimens, poor responses to treatment, childhood onset, a family history of mental disorders, excitability, obsessive–compulsive symptoms, lack of sleep, anxiety and obesity.

Treatment-related risk factors for readmission included longer initial hospitalization, poor adherence to medication, frequent changes in treatment regimens and poor responses to the modified electroconvulsive therapy (MECT). Among children and adolescents with autism, a longer initial hospitalization of over 15 days was associated with higher 31-day and 1-year unplanned readmission rates compared to a stay of below 7 days (33). The duration of hospitalization might also be related to treatment difficulties. This pattern is illustrated in an earlier study of patients with schizophrenia: during the second and third hospitalizations, the length of stay increased, medication dosages were escalated, while treatment effectiveness progressively declined (26). A study of 81 children and adolescents with schizophrenia revealed that poor adherence to medication (RR = 5.21, 95% CI: 2.00–13.63) and a high number of changes in the treatment regimen (RR = 5.10, 95% CI: 1.97–13.24) predicted an increased probability of rehospitalization within 3–5 years (28). Another study reported that 77.8% of the patients who were readmitted within half year experienced discontinuation or reduction of medication on their own (22). MECT may be an effective treatment for bipolar disorder, particularly for improving long-term outcomes. Among children and adolescents with bipolar mania, although no significant difference in readmission rates was observed, those who received a combination of MECT and medication demonstrated a significantly better cumulative survival curve than those treated with medication alone (35). In another study examining the association between response to MECT and readmission risk, a significant relationship was found in youth with bipolar disorder (readmission rates in responders vs. nonresponders were 19.5% vs. 34.5%), but not among youth with schizophrenia or depression (34).

Disease characteristic-related risk factors for readmission included onset age, family history, and specific symptoms. Regardless of diagnosis, patients with childhood-onset disease (onset age ≤ 13 years) had a higher readmission rate than those with adolescent–onset disease (onset age 13–18 years) (23, 24). One large-scale retrospective study examined the associated factors at contextual, hospital and individual levels regarding readmission and found that the number of general practitioners within cities was associated with reduced risk of unplanned readmissions (8). Among adolescents with depression, a family history of mental disorders, inadequate sleep (i.e., <9 hours for adolescents aged 12–15 years, <8 hours for adolescents aged 16–18 years), and a one-point higher self-rating anxiety scale score increased the likelihood of 1-year readmission by 4.44 times (95% CI: 1.36–14.49), 2.76 times (95% CI: 1.01–7.53), and 4.30 times (95% CI: 1.58–11.73), respectively (32). Among children and adolescents with schizophrenia, higher levels of excitability and the absence of obsessive–compulsive symptoms significantly increased the risk of readmission by 2.47 times (95% CI of RR: 1.22–5.00) (28) and 3.33 times (95% CI of OR: 1.30–9.56) (30), respectively.

Obesity was a comorbidity related to an elevated rate of rehospitalization among adolescents. Specifically, a BMI ≥ 24 kg/m^2^ increased readmission by 3.11 times (95% CI of OR: 1.18–8.22) for depressed patients after one year of discharge (32).

One study conducted among adolescents with major depressive disorder suggested that support from family, teachers, and friends was associated with a lower risk of rehospitalization one year after discharge (32).

### 3.4 Intervention programs to reduce psychiatric readmission

Five interventional studies were incorporated into the narrative review. Three focused on reducing psychiatric readmission among children and adolescents, while one aimed to reduce parental emotional symptoms, and the other targeted youth stigma. A narrative synthesis of the interventional studies is provided in **Table 3**.

**Table 3:** Characteristics of included interventional studies targeting psychiatric readmission.

Reference (Author, year)	Language	Article type	Study design	Research period	Sample characteristics	Main findings related to readmission
Zhi, 2012 (36)	Chinese	Original article	Randomised controlled trials	NA	96 adolescents with internet addiction	Adolescents in the intervention group received a one-year multidimensional intervention in addition to regular care. The intervention included psychological support, disease and treatment-related education, nutritional interventions, and outpatient support. The readmission rate was significantly lower in the intervention group than in the control group after 1-yr (11.4% vs. 32.5%).
Liu, 2013 (37)	Chinese	Original article	Intervention	2012-2013	105 parents of youth with multiple psychiatric admissions who presented anxiety and depressive symptoms	Supportive psychological interventions and relaxation training reduced depression and anxiety symptoms in parents.
Ou, 2013 (38)	Chinese	Original article	Intervention and follow-up (3 months)	2008-2011	36 school-age children with psychogenic diseases, aged 6-14-yrs	The 36 children with psychogenic illnesses received continuous and holistic nursing care. Responsible nurses assessed the children and their families through in-depth interviews to identify the causes or triggers, and then implemented family-centred psychological nursing care to address them. After 10 to 30 days of inpatient treatment, 30 cases were recovered, 29 cases had no relapse after discharge, 1 case relapsed 2 months after discharge due to a trigger but recovered after 16 days of readmission, and 7 cases improved.
Yang, 2017 (35)	Chinese	Original article	Randomised controlled trials	2012-2014	42 patients with bipolar mania aged below 18-yrs	Pediatric patients with bipolar mania were randomized to receive either MECT (8 times) combined with drug therapy (Group A) or drug therapy (Quetiapine and Lithium) alone (Group B). The readmission rates for Group A were 5%, 5%, and 10%, compared to 18.2%, 27.3%, and 31.8% for Group B, during the 6-month, 12-month, and 2-year follow-ups. Although no significant difference in readmission was observed, Group A demonstrated a significantly better cumulative survival curve than Group B (log-rank χ²= 9.140, P < 0.01). The incidence of adverse events did not differ significantly between the groups.
Sun, 2017 (39)	Chinese	Original article	Randomised controlled trials	2012-2015	66 psychiatric inpatient children and adolescents diagnosed with MDD aged 13-18-yrs old	Patients readmitted to psychiatric ward had a stronger stimga than those who were first admitted. Cognitive Behavioural Therapy (CBT) significantly reduced stigma in depressed patients.

One randomized controlled trial (RCT) conducted among patients with bipolar mania demonstrated that the combination of pharmacological treatment and MECT reduced the risk of readmission over 2 years, thereby improving survival rates, compared with pharmacotherapy alone (35).

Another RCT which focused on adolescents with internet addiction who underwent a multidimensional intervention showed effectiveness in reducing readmission. The interventions included psychological support, education on illness and treatment, nutritional interventions, and outpatient support. The 1-year readmission rate was significantly lower in the intervention group than in the control group (11.4% vs. 32.5%) (36).

A preliminary study combining psychological nursing and family-centered counseling explored the beneficial effects of psychological interventions during hospitalization on the prognosis of children with psychogenic illnesses. The results indicated that after 10–30 days of comprehensive psychological intervention, 83% (30/36) of children have recovered by discharge, with only one experiencing recurrence within three months of discharge. Two major limitations of the study were the small sample size (i.e., only 36 families were included) and the absence of a control group (38).

Adolescents readmitted to psychiatric wards experienced greater stigma than those who were admitted for the first time. A randomized controlled trial revealed that cognitive behavioral therapy (CBT) significantly reduced stigma in depressed adolescent inpatients (39).

Parents of children and adolescents with repeated psychiatric admissions (at least 3 times) could also experience tremendous psychological distress. A six-week program of supportive psychological interventions and relaxation training were found to be effective in reducing depression and anxiety symptoms in parents (37).

## 4 Discussions

### 4.1 Main findings

Psychiatric readmission rates among children and adolescents vary widely, depending on the diagnosis, follow-up duration and study characteristics. Depression was associated with lower readmission rates, whereas patients with schizophrenia had higher rates of readmission. As the duration of follow-up increased, the likelihood of readmission also increased. Risk factors for readmission included treatment challenges, younger age at onset, family history, and complex disease presentations. Conversely, strong social support emerged as a significant protective factor against readmission. Very little effort has been made to reduce psychiatric readmissions for children and adolescents in China. The quality of the studies varied greatly, indicating a need for larger controlled trials and standardized methodologies.

The short-term readmission (i.e., <1year) rate was 8.6%, lower than the long-term readmission (i.e., >1 year) rate of 20.9% in the current review. Early and long-term readmission might be associated with different factors. For example, evidence from studies of adults with severe mental illness indicates that early readmissions are often related with clinical factors (e.g., symptom severity, comorbid personality disorder), treatment factors (e.g., insufficient treatment, discharge against medical advice), and lack of social support (40). In contrast, long-term readmission is less predictable and may be more closely tied to system factors such as the availability and intensity of community support (41). Less evidence was available among pediatric psychiatric inpatients, though certain clinical features have been linked to readmissions (e.g., aggression and suicidallity) (42). Moreover, the early post-discharge period (i.e., within one year) is a critical window associated with elevated all-cause mortality (43). Therefore, stage-specific strategies are essential to reduce readmission. In the immediate post-discharge period, targeted early interventions should focus on intensive clinical management for high-risk individuals, whereas long-term strategies must prioritize strengthening systemic and community-based care with enhanced support and monitoring to sustain recovery.

The readmission among children and adolescents in heterogeneous diagnostic groups seemed to be lower than that in the adult group in China (8). The relatively lower readmission rate for children and adolescents might be because they are readmitted to adult psychiatric facilities after a recurrence for various reasons, such as aging or a shortage of pediatric psychiatric beds. Additionally, psychiatric disorders in children and adolescents may be episodic. A previous study reported that approximately half of these episodes have a duration of less than six months, and their mental health problems may not persist or reoccur in adulthood (12). It is also possible that psychiatric disorders emerging during childhood and adolescence differ from those in adults, with emotional disorders typically occurring earlier than psychotic disorders. It is well documented worldwide and across age groups that patients with more severe symptoms and psychotic disorders may have an increased likelihood of readmission (1, 7, 44). When only patients with depression were compared, the readmission rate among young people was higher than that among adults (31).

Compared with children and adolescents in Western countries, the overall readmission rate is higher in China. In a meta-analysis, the overall readmission rate, regardless of diagnosis, was 13.2% (1), whereas in the current review, it was 18.6%. A similar situation was observed for youth with schizophrenia (i.e., 27.6% vs. 12.1%), while the readmission rate for depression could be comparable in China to that in other countries (i.e., 4.9%-17.6% vs. 10.1%). This diagnosis specified variability could be attributed to systemic factors within China’s healthcare system. Specifically, the historically limited availability of pediatric psychiatric inpatient resources has led to a prioritization of beds for patients with more severe conditions such as schizophrenia and bipolar disorders, which typically manifest as behavioral disruptions. Conversely, disorders characterized by internalizing symptoms such as depression in children and adolescents are easily overlooked because of their subtlety and stigma surrounding mental health issues in China, resulting in lower admission and readmission rates. Additionally, while high readmission rates for schizophrenia were reported in publications before 2020, two recent publications reported lower rates. In contrast, readmission rates for mood disorders are mainly reported in publications after 2020. This pattern might suggest a transition in the healthcare system in response to shifting inpatient service needs, as the most prevalent psychiatric disorder reported in earlier publications was schizophrenia (45, 46), whereas in recent years, mood disorders have been the most reported in the pediatric inpatient population (23, 47).

A clear relationship between the clinical severity of the disease and psychiatric readmission risk was also found in Chinese children and adolescents, which is consistent with other studies (1, 7). The severity of the disease can be reflected in multiple aspects. From the perspective of family history, young patients with a family history of mental illness not only have a genetic predisposition but also face greater treatment difficulty (48, 49). From the perspective of the age of disease onset, the current review revealed some evidence suggesting a negative impact of early age of onset on long-term social outcomes, as indicated by repeated hospitalizations. The prognosis of early-onset mental illness is not always consistent across different contexts. For example, age at disease onset does not predict long-term social functions in populations with early-onset schizophrenia (50). An earlier age of onset of bipolar disorder is associated with increased comorbidity but not with other indicators of severity or treatment resistance (e.g., psychotic symptoms) (51). Nevertheless, major depressive disorder tends to be more chronic when it begins during adolescence (52). Accordingly, future research is warranted to investigate the pathways and potential mediators that connect early onset age to long-term prognosis across various diagnoses.

Comorbid physical conditions, such as abnormal blood pressure, lipid profiles and thyroid function, are often associated with increased risks of psychiatric readmission in adult patients (53, 54). Moreover, depressed adolescents with comorbid obesity are at an elevated risk of rehospitalization (32), highlighting the importance of monitoring weight and metabolic functions when designing interventions to reduce readmissions. Furthermore, the presence of comorbid physical illnesses, especially metabolic-related diseases, may be associated with many determinants, such as the emotional symptoms themselves, adverse drug effects, and psychosocial factors. Therefore, it is important to identify the potential causes of physical comorbidities in children and adolescents with mental illness from multiple perspectives to further reduce disease relapse and readmission.

Regarding interventions to reduce readmission among children and adolescents, the evidence is very limited. Five interventional studies were identified, three of which focused directly on how to reduce readmissions. Two RCTs successfully reduced the readmission rate, one was conducted among young patients with internet addiction using psychotherapy, and the other was conducted among patients with bipolar mania using pharmacological treatment combined with MECT. Regarding MECT treatment, although it is acknowledged as a viable option for adolescents with severe, life-threatening symptoms (e.g., suicide attempt and catatonia) by the guidelines for the bipolar disorder, its use in this population remains cautious. This is due to a limited evidence base, with some reports indicating a longer remission course compared to adults (55), and restricted access in many remote or resource-limited settings. Despite the limited number of studies, one study aimed to provide emotional support for parents of inpatient children and adolescents with mental health problems. Family-related factors are closely related to the clinical course of children and adolescents. For example, harsh parental discipline and increased parent–child conflict can impact the risk of readmission (56), whereas positive engagement of family members can improve treatment adherence, reduce triggers for recurrence, and enhance prognosis (32, 38). Research in adults has also confirmed that family interventions can effectively improve the prognosis of individuals with mental disorders (57). Our review suggests that researchers, clinicians, and policymakers should accelerate research to provide more efficient interventions tailored to the local context for children and adolescents with urgent hospitalization needs to facilitate their recovery and improve social functioning.

### 4.2 Limitations and research agenda

Based on the results of this review, several research gaps were identified. First, the methodological quality of the included studies varied considerably. Most studies relied on small sample sizes and used self-developed assessment tools, which typically have limited validity. The predominant use of retrospective designs, inconsistent definitions of the readmission observation period, and the failure to distinguish planned from unplanned readmissions complicate the interpretation of findings, as the factors influencing readmission likely differ across time frames. For instance, short-term readmission (e.g., within 30 days) may reflect the quality of inpatient and transitional care, whereas longer-term readmission (e.g., one year or more) may be more closely associated with disease characteristics, rehabilitation outcomes, and social support. Future research should employ larger samples, prospective designs, and more rigorous methodologies to better understand and address psychiatric readmission among children and adolescents in China.

Second, several critical components at the social level related to the psychiatric readmission of children and adolescents remain unevaluated in China. For example, few studies have explored the impact of sociodemographic factors on readmission. Children and adolescents from socioeconomically disadvantaged backgrounds are two to three times more likely to develop mental health problems (58). In adults, urban residents and unemployed patients with either public or private medical insurance are more likely to be readmitted (9, 31). Therefore, it is expected that children and adolescents in areas with slower socioeconomic development are at increased risk of readmission and require more attention and resources. In addition to socioeconomic status, stigma and social discrimination could also influence the use of mental health services among children and adolescents. In Brazil, family stigma toward mental illness may contribute to increased readmission rates among individuals with psychiatric disorders (59). Moreover, individuals with lower socioeconomic status tend to hold more stigma toward people with mental illness (60). Therefore, when evaluating the mental health service system for children and adolescents, it is essential to understand how economic and social factors influence disease occurrence, development, and the accessibility and quality of resources.

Third, this scoping review revealed that the factors influencing readmission at the health service level are inadequately documented. Key areas lacking comprehensive data include patient satisfaction with inpatient services, discharge against medical advice, and the percentage of patients receiving predischarge planning and postdischarge aftercare. Moreover, there is a lack of evidence regarding successful interventions in China aimed at reducing the readmission rate for children and adolescents and promoting their social reintegration after discharge from psychiatric hospitalization. In-hospital treatment is usually considered a prior option for severe cases, while a number of studies have shown that adolescents with a history of previous hospitalization have a significantly greater risk of readmission than do those without a history of previous hospitalization (1). Compared with hospitalization, home treatment (61, 62) and intensive community treatment (63) can provide equivalent immediate and long-term therapeutic effects and may help avoid additional problematic issues such as disrupted social support and interrupted education (64). Hence, intensive outpatient care for severe cases could be an effective strategy for mitigating the additional risks associated with inpatient treatment. Although alternative care services have been developed in some countries, due to differences in cultural and healthcare systems, these models cannot be directly applied in China. To improve the long-term prognosis of children and adolescents with severe mental health problems, policymakers should consider improving current healthcare services. This might include establishing procedures to ensure clear responsibilities and transparency at all levels of medical institution, optimizing information technology and referral mechanisms, and enhancing collaboration with patients, families, and professionals (65).

## 5 Conclusions

Our findings hold significance for policymakers regarding the enhancement of the mental health services system for children and adolescents in China, especially given the growing attention being paid to child and adolescent mental health, which includes the establishment of more pediatric psychiatric clinics and wards. On the positive side, more children and adolescents with severe conditions are now able to receive timely and necessary treatment. As a result of the expansion of inpatient services, there is an anticipated rise in the number of children and adolescents receiving inpatient treatment, who may also face the challenge of reintegrating into society after discharge. However, significant limitations remain, such as the absence of objective criteria for assessing the indications for inpatient treatment and the lack of corresponding support services pre- and post- hospitalization. Hence, standardized and continuous care across the entire hospitalization process is essential for reducing admissions and readmissions, and ultimately for improving the long-term outcomes of children and adolescents with mental health problems.

## Data Availability

The original contributions presented in the study are included in the article/**Supplementary Material**. Further inquiries can be directed to the corresponding author/s.
